# The coevolutionary mosaic of bat betacoronavirus emergence risk

**DOI:** 10.1093/ve/vead079

**Published:** 2023-12-20

**Authors:** Norma R Forero-Muñoz, Renata L Muylaert, Stephanie N Seifert, Gregory F Albery, Daniel J Becker, Colin J Carlson, Timothée Poisot

**Affiliations:** Département de Sciences Biologiques, Université de Montréal, 1375 Avenue Thérèse-Lavoie-Roux, Montréal (Québec) H2V 0B3, Canada; Québec Centre for Biodiversity Sciences; Molecular Epidemiology and Public Health Laboratory, School of Veterinary Science, Massey University, New Zealand; Paul G. Allen School for Global Health, Washington State University, Pullman, WA, United States; Department of Biology, Georgetown University, Washington, DC, USA; Department of Biology, University of Oklahoma, Norman, OK, USA; Department of Biology, Georgetown University, Washington, DC, USA; Center for Global Health Science and Security, Georgetown University Medical Center, Washington, DC, USA; Department of Microbiology and Immunology, Georgetown University Medical Center, Washington, DC, USA; Département de Sciences Biologiques, Université de Montréal, 1375 Avenue Thérèse-Lavoie-Roux, Montréal (Québec) H2V 0B3, Canada; Québec Centre for Biodiversity Sciences

**Keywords:** bats, betacoronavirus, disease ecology, geographic mosaic theory of coevolution

## Abstract

Pathogen evolution is one of the least predictable components of disease emergence, particularly in nature. Here, building on principles established by the geographic mosaic theory of coevolution, we develop a quantitative, spatially explicit framework for mapping the evolutionary risk of viral emergence. Driven by interest in diseases like Severe Acute Respiratory Syndrome (SARS), Middle East Respiratory Syndrome (MERS), and Coronavirus disease 2019 (COVID-19), we examine the global biogeography of bat-origin betacoronaviruses, and find that coevolutionary principles suggest geographies of risk that are distinct from the hotspots and coldspots of host richness. Further, our framework helps explain patterns like a unique pool of merbecoviruses in the Neotropics, a recently discovered lineage of divergent nobecoviruses in Madagascar, and—most importantly—hotspots of diversification in southeast Asia, sub-Saharan Africa, and the Middle East that correspond to the site of previous zoonotic emergence events. Our framework may help identify hotspots of future risk that have also been previously overlooked, like West Africa and the Indian subcontinent, and may more broadly help researchers understand how host ecology shapes the evolution and diversity of pandemic threats.

Disease emergence is complex, and is driven not only by animal–human contact, but also by the underlying evolutionary dynamics in viral reservoirs (Plowright et al. 2017). Although host richness is often used as a superficial proxy for spillover risk (Anthony et al. 2017; Ruiz-Aravena et al. 2022; Sánchez et al. 2022), these approaches oversimplify the relevant interspecific heterogeneity in immunology, behavior, and other traits, and therefore overlook unique host pools that allow for the rapid evolution of highly divergent viruses (Agosta, Janz, and Brooks 2010). In the case of generalist pathogens like betacoronaviruses, there is a conceptual and empirical support to the idea that these community-level mechanisms are even more important (Power and Mitchell 2004), particularly given that cross-species transmission may, as a rule, structure viral evolution more than codivergence with hosts (Geoghegan, Duchêne, and Holmes 2017). This creates a disconnection between coevolutionary theory and most existing ecological frameworks for mapping spillover risk.

The geographic mosaic theory of coevolution (GMTC) attempts to explicitly connect microevolutionary dynamics to the macroecology and biogeography of symbiotic interactions (Thompson 2005). The GMTC posits that coevolutionary processes among pairs (Thompson 1994) or complexes (Janzen 1980) of species are structured in space by the rippling effects of abiotic conditions onto evolutionary mechanisms, giving rise to fragmented systems with different ecologies over large spatial extents (Price 2002). The GMTC predicts a spatial fragmentation of coevolutionary dynamics under the joint action of three processes (Gomulkiewicz et al. 2007): coevolutionary hot- and coldspots, which appear when the intensity of *interaction* (in terms of reciprocal fitness consequences) varies spatially; selection mosaics, wherein the intensity of *selection* varies across space, driven by both the biotic complexity of the community (locally diverse hosts and viruses are more biotically complex) and the local favorability of the environment (Thrall et al. 2007); and trait remixing, which occurs when coevolutionary dynamics change when community-level *functional traits* change through meta-community dynamics.

Here, we apply the GMTC to explore and explain the global biogeography of betacoronaviruses, the group that includes SARS-associated coronavirus (SARS-CoV), Middle East respiratory syndrome coronavirus (MERS-CoV), and SARS-CoV-2. In their bat reservoirs, coronaviruses evolve through a mix of host jumps, recombination among disparate lineages, and, to a lesser degree, codivergence with their hosts (Anthony et al. 2017)—a mix of mechanisms that creates a complex and nonlinear relationship between host diversity and viral emergence. Working from a recently published database of bat hosts of betacoronaviruses, we test whether spatial structure in bat–betacoronavirus coevolution is identifiable at a global scale. Aiming to explain these patterns, we develop a generalized framework for applying the GMTC to host–virus interactions, with a specific emphasis on the potential to create independent coevolutionary dynamics (and therefore, spatial fragmentation in risk) through heterogeneity. We develop a trivariate risk assessment system that connects each GMTC mechanism to a quantifiable aspect of host–virus interactions: (1) viral sharing rates in host communities, representing the strength of potential interaction between viruses and any one host (i.e. places where viruses undergo constant host switching may be coevolutionary coldspots); (2) the phylogenetic diversity of hosts, as a proxy for variation in the immunological mechanisms that antagonize viruses (i.e. the selection mosaic); and (3) the local uniqueness of the bat community, representing the potential for viruses to be exposed to novel host traits (e.g. variation in receptor sequences). Together, we argue that these can be used to identify and map the evolutionary drivers that—in conjunction with transmission processes (e.g. viral prevalence in reservoirs and animal–human contact rates)—determine disease emergence risk.

## Results and Discussion

### Bat and betacoronavirus biogeography are broadly consistent

**Figure 1.**
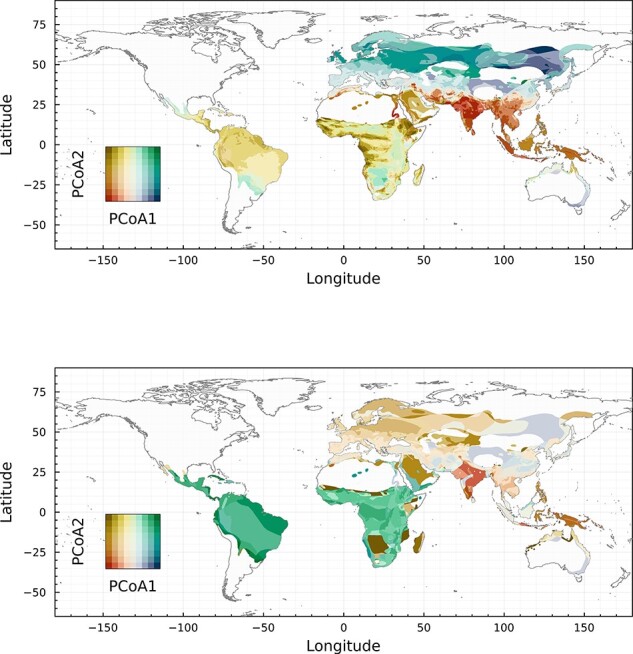
**Bat and betacoronavirus biogeographic regions**. Phylogeography of bats (top) and viruses (bottom) is categorized based on an analysis of bat distributions, paired with bat or virus phylogeny. The different colors show tendencies to separate alongside the first two components of a PCoA. Note that the PCoA for the bats and viruses are independent, and so cannot be compared directly—that being said, the fact that different regions cluster in the same way across maps be directly compared.

Most previous work has assumed that the presence or richness of key groups of bat hosts is predictive of coronavirus diversity (Anthony et al. 2017; Ruiz-Aravena et al. 2022). Projecting bat and betacoronavirus phylogeny over space (Fig. 1), we find support for the idea that bat community assembly is directly responsible for a global mosaic of viral evolution. The distinct groupings (represented by different colors, symbolizing positions in a subspace formed by the first two phylogenetic principal components) are essentially equivalent between the two groups, and can be coarsely delineated as (1) south and southeast Asia; (2) east Asia (including northern China), west Asia, and the Mediterranean coast; (3) Eurasia above a northing of 40; and (4) Africa and Latin America. In some cases, this diverges from expectations about coronavirus biogeography: for example, previous work has rarely flagged India as a region of interest, but for both bats and betacoronaviruses, the subcontinent falls into the same regions as the southeast Asian peninsula (and indeed, the region is home to known bat hosts of multiple betacoronavirus subgenera, including nobecoviruses, sarbecoviruses, and merbecoviruses) (Ruiz-Aravena et al. 2022).

Overall, these results suggest that the boundaries of bat and betacoronavirus biogeographic regions are broadly consistent at a global scale; perfect matching between the biogeographic regions would have indicated that the signal of virus distribution is fully predicted by bat hosts ranges. Areas for which the biogeographic regions for bats and betacoronaviruses differ are primarily (1) southeast Asia and southern China, and (2) the Arabian Peninsula, which are both regions where zoonotic transmission has been documented (potentially driving a unique level of viral sampling effort that generates these patterns). These spatially limited mismatches nonwithstanding, the large level of congruence may be surprising, given that cross-species transmission may play a stronger role in coronavirus diversification than cospeciation (Anthony et al. (2017)—a property that would theoretically allow for substantial broad divergence in their biogeography. However, host jumps at the family level or higher are relatively rare and significant events in coronavirus evolutionary history (Anthony et al. 2017; Latinne et al. 2020); as a result, the mosaic of betacoronavirus phylogeography is assembled from a set of overlapping smaller coevolutionary systems, superimposed in space and filtered by the importance of different subgroups in local host communities. For example, the most speciose and cosmopolitan family of bats, the vesper bats (Vespertilionidae), are considered the primary hosts of the subgenus *Merbecovirus* (MERS-like viruses) (Latinne et al. 2020; Ruiz-Aravena et al. 2022); but in the Americas, where merbecoviruses are the only lineage present, they have only been found in other bat taxa (e.g. Molossidae, Phyllostomidae) (Brandão et al. 2008; Anthony et al. 2013; Góes et al. 2013, 2016). At the coarsest scale, these heterogeneities are lost, and betacoronavirus biogeography tracks the deep rifts in bat evolutionary history—but within broad regions, the component coevolutionary systems may have very different dynamics.

### Hotspots of bat and betacoronavirus biodiversity are distinct

**Figure 2.**
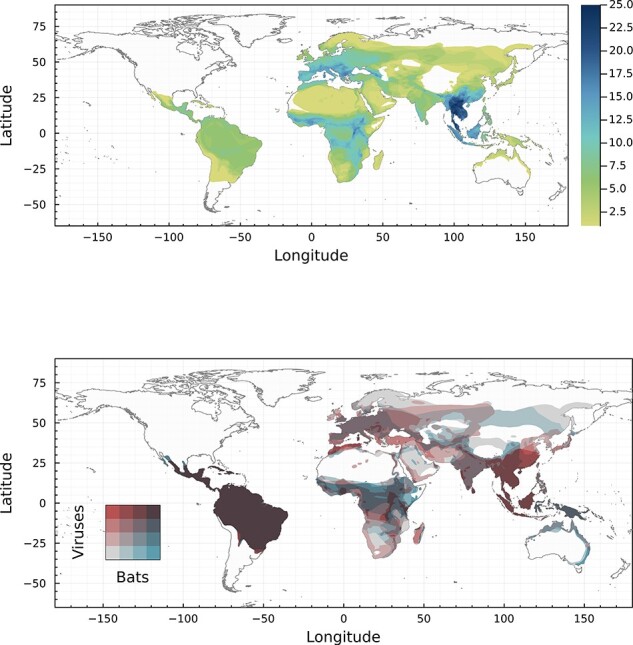
**Bat and betacoronavirus diversity**. Top panel: diversity of known bat hosts of betacoronaviruses in our dataset. This map shows that the region with the largest number of possible hosts is South-Eastern Asia. Bottom panel: congruence between the *evolutionary* distinctiveness of the hosts (grey to blue) and the viruses (grey to red). Darker areas have higher combined evolutionary distinctiveness for the entire bat–virus system.

Bats, the second most diverse groups of mammals, are found worldwide; gradients in their species richness generally track broader patterns of mammal diversity (Tanalgo, Oliveira, and Hughes 2022) with a striking Neotropical hotspot (especially in the Amazon basin) and a secondary hotspot centered in Indochina. These hotspots of bat diversity are generally presumed to be hotspots of viral adaptive radiation, and therefore areas of concern for human health (Anthony et al. 2017; Olival et al. 2017). However, the hotspots of known bat betacoronavirus hosts show a distinct pattern, with primary hotspots (both in terms of area and higher values) of host richness situated in southeast Asia, parts of southern Europe, and to a lesser extent parts of Africa in the −25 to 0 range of latitudes (Fig. 2; top). Although hundreds of species likely host undiscovered betacoronaviruses, machine-learning predictions have suggested that these undiscovered reservoirs should follow the same diversity gradient (Becker et al. 2022). In principle, these hotspots of locally diverse, virus-rich bat communities should drive more adaptive diversification in their viruses.

However, we find that the global pattern of betacoronavirus phylogenetic distinctiveness is quite distinct from both bat host richness and phylogenetic distinctiveness (Fig. 2; bottom). In contrast to the sparsity of Neotropical betacoronavirus hosts, South and Central America have the most evolutionary distinct hosts *and* viruses, followed by secondary hotspots in southeast Asia and the Rift Valley region have mostly distinct viruses. Some degree of sampling bias may contribute to these patterns: for example, the Neotropics are one of the places where the fewest bat betacoronavirus sequences have been generated (Boni et al. 2020; Temmam et al. 2022^;^  Worobey et al. 2022), resulting in a sparser phylogenetic tree, and artificially inflating distinctiveness; conversely, disproportionate research effort in eastern China (Cohen et al. 2022) may have led to a more complete inventory of the local diversity of coronaviruses, again inflating these metrics relative to underlying patterns. Even accounting for these potential biases, though, there is obvious heterogeneity in betacoronavirus evolutionary distinctiveness that is distinct from overall bat diversity.

Overall, these patterns recapitulate the evolutionary history of both the order Chiroptera and the genus *Betacoronavirus*. Horseshoe bats (Rhinolophidae) include the reservoirs of the SARS-like viruses (subgenus *Sarbecovirus*), the group of pandemic threats that have been of the greatest interest to researchers (Latinne et al. 2020) (and so have been sampled most intensively) (Cohen et al. 2022). The hotspots of host richness and viral diversity in Southeast Asia—both of which are disproportionately high, considering the global landscape of bat species richness—are almost entirely driven by viral adaptive radiation through host switching within this clade (Becker et al. 2022; Ruiz-Aravena et al. 2022). In contrast, the Neotropical hotspot of viral distinctiveness is driven by isolation by host vicariance. Out of the four main groups of betacoronaviruses, only merbecoviruses have been found in animals in the Americas—an introduction that is generally presumed to be ancient (Olival et al. 2020^;^  Ruiz-Aravena et al. 2022). While comparatively understudied, New World merbecoviruses have been found in the ghost-faced bats (Mormoopidae), Neotropical leaf-nosed bats (Phyllostomidae), and free-tailed bats (Molossidae) (Brandão et al. 2008; Anthony et al. 2013; Góes et al. 2013^,^  2016). The former two groups and a clade of the latter are endemic to the Neotropics, while the explosive adaptive radiations of the phyllostomids are responsible for the hotspot of bat diversity in the Amazon (Ammerman, Lee, and Tipps 2012). Together, these clades of New World bats play host to a distinct regime of betacoronavirus coevolution.

Our approach is potentially limited by sampling bias: key hotspots identified by our model have, indeed, been sampled intensely following major zoonotic emergence events. In these areas, more betacoronavirus hosts will have been discovered, leading to higher overall diversity and potentially higher sharing. Similarly, hotspots of evolutionary uniqueness—as in the Arabian Peninsula—could reflect much broader lineages that have only been sampled in focal areas for public health. While the discovery of new branches of bat–betacoronavirus coevolution is certainly likely, and might change some of the observed patterns, our framework is likely to be fairly robust: the 126 hosts in our study capture nearly 10 per cent of global bat diversity, and the underlying evolutionary patterns they represent are much less sensitive to new information than any inferences about viral evolution.

### Coevolutionary regimes structure evolutionary potential for zoonotic emergence

The existence of well-defined cophylogenetic regions suggests that the bat–betacoronavirus system is spatially fragmented enough to create divergent coevolutionary trajectories; in turn, this coevolutionary mosaic may alter the risk of zoonotic emergence. These ideas are, respectively, supported by the existence of hotspots of viral uniqueness and the diverse origins of human betacoronaviruses. Together, this framework points to a predictable relationship between host community structure and coevolutionary pressure: phylogeographic structure in bat hosts (and their diverse immune strategies) (Banerjee et al. 2020) creates a landscape of selective pressure; the trajectory of viruses’ coevolutionary response is, in turn, constrained by their opportunities for either specialization or diversification through host jumps and recombination.

**Figure 3.**
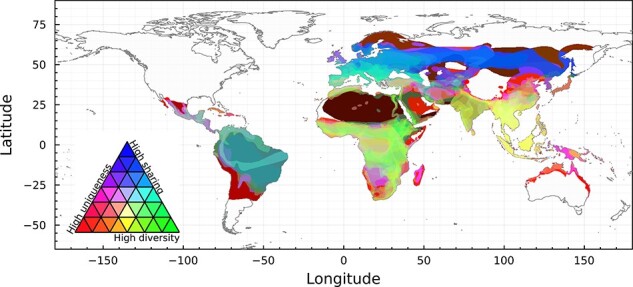
**Trivariate additive mapping of the components of risk**. Viral sharing runs from yellow (low) to blue (high); host phylogenetic diversity runs from pink (low) to high (green); and host compositional uniqueness runs from cyan (low) to red (high). The GMTC suggests that the highest evolutionary potential for emergence exists in unique and diverse host communities with low viral sharing, i.e. pixels around yellow. All components within bat host ranges are scaled in brightness so that a pixel with no sharing, no phylogenetic diversity, and no compositional uniqueness would be black, and a pixel with maximal values for each would be white. The individual layers that compose this figure are given in Supplementary Material.

Based on the geographic mosaic theory of coevolution, we developed a trivariate map of coevolutionary pressure (Fig. 3): (1) *host phylogenetic diversity*: a high diversity of evolutionary histories should expose viruses to more variation in host immune traits; (2) *host community uniqueness*: exposure to greater host trait heterogeneity can drive viral diversification, and coevolving with more unique host communities should create more unique branches of viral evolution; and (3) propensity for *viral sharing*: frequent cross-species transmission may act as a buffer on selective pressure, while lower rates of exchange may enable more simultaneous trajectories of viral specialization to coexist within a given community. We combine global maps of all three to generate a map of coevolutionary regimes, where close colors represent similar risks, and paler pixels represent overall higher risk (see ‘Methods’ section). We find that these regions do not neatly overlap with those defined in Figs. 1 or 2, reinforcing the notion that local-scale coevolutionary mosaics can form within cophylogenetic regions.

**Figure 4.**
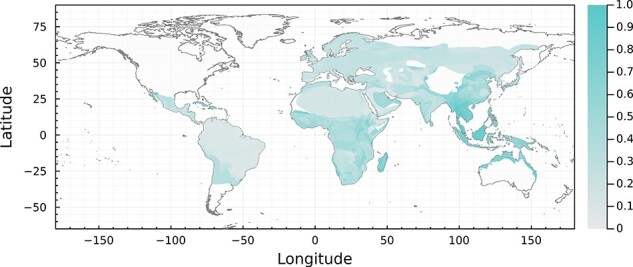
**Evolutionary potential for zoonotic emergence of bat-origin betacoronaviruses**. Risk is a composite measure of the color value and angular distance to the yellow hue in Fig. 3 (see ‘Methods’ section). Darker pixels represent areas where the coevolutionary mechanisms are likely to introduce a strong risk of emergence.

The greatest evolutionary potential for zoonotic emergence exists where pathogen pools have a high genetic diversity and high propensity for cross-species transmission. In our framework, emergence risk is therefore maximized under higher phylogenetic diversity (viruses are exposed to different host clades), higher host uniqueness (viruses are experiencing novel, heterogeneous host traits combinations), and low to medium viral sharing (host–virus pairs can coevolve independently, but divergent viruses may still have opportunities for recombination). In Fig. 3, this corresponds to yellow areas (dynamics dominated by low viral sharing, with equal contributions of selection mosaics and trait remixing; southeast Asia, and the Indian sub-continent), green–yellow areas (dynamics with low viral sharing but dominated by the selection mosaic effect of host diversity; sub-Saharan Africa), and red–yellow areas (dynamics with low viral sharing but dominated by trait remixing in host communities; the Middle East). Translating this axis of variation back into a univariate risk map (Fig. 4) highlights that this evolutionary landscape has a striking correspondence to regions where zoonotic betacoronaviruses have previously emerged. Our findings align with predictions regarding the spatial location of cross-species transmission. These locations not only pose a potential risk of viral jumps that could endanger human health but also provide valuable information for monitoring wildlife health. This could guide us to determine where and what measures to implement for effectively monitoring wildlife and human betacoronavirus outbreaks before they escalate to critical levels. Nevertheless, there are actually very few documented cases of emergence events, and similarities could be some degree of coincidental.

Compared to approaches that map emergence risk based only on the number of known bat hosts of betacoronaviruses, our framework suggests regions where high viral sharing dominates coevolutionary dynamics—such as Latin America, or Eurasia above a northing of 30—would pose less of a relative risk of zoonotic emergence. Nevertheless, areas of high host uniqueness coupled with high viral sharing (red-to-pink in Fig. 3) could create hotspots facilitated by viral codivergence. Our framework identifies Madagascar, where most bat species are endemic following evolutionary divergence from sister species in both African and Asian continents (Shi et al. (2014) as one such hotspot; interestingly, a recent study (Kettenburg et al. 2022) reported a novel and highly divergent lineage of nobecoviruses from Madagascar-endemic pteropid bat species (*Pteropus rufus* and *Rousettus madagascariensis*), again supporting the predictive power of the coevolutionary framework.

### Human landscapes filter the geography of emergence risk

**Figure 5.**
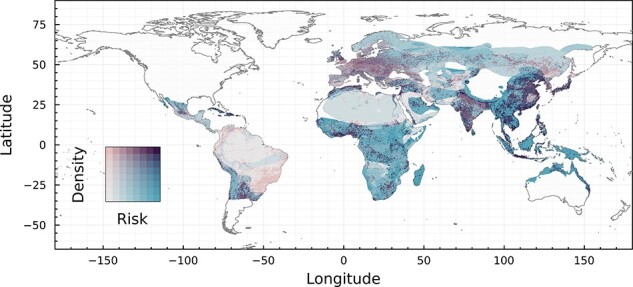
**Overlap between evolutionary potential and ecological opportunity for zoonotic emergence**. Overlap of the percent of each pixel occupied by urbanized structures, representing the degree of settlement, on the spillover risk map (where the risk comes only from wildlife, and ignores multi-hosts chains of transmissions including non-bats hosts). Darker pixels correspond to more risk, in that the GMTC-derived risk of Fig. 4 is high *and* the pixel is densely occupied by human populations.

The relationship between the underlying pathogen pool and emergence risk is mediated by both human–wildlife interfaces (the probability of spillover) and opportunities for onward horizontal transmission (the probability that spillovers become epidemics) (Plowright et al. 2017). It must be noted that the assesment of risk based on the GMTC mechanisms does not account for human presence; for this reason, it represents ‘potential’ level of risk, which must be re-evaluated in the light of human presence. As a proxy for both, we finally overlaid the risk component from the composite map (see above) with the proportion of built land, as a proxy for a mix of habitat disturbance, potential for bat synanthropy or contact with bridge hosts like livestock (Cui, Li, and Shi 2019; Rulli et al. 2021) and human population density and connectivity (Hassell et al. 2017; Plowright et al. 2017; Muylaert et al. 2022) (Fig. 5). Accounting for these factors, most of South America and Europe are at comparatively lower risk, as—although densely populated—settlements tend to be in areas with lower potential risk. Conversely, regions like Malaysia and the northern coast of Australia have a high evolutionary risk component, but should represent a relatively lower effective risk due to low human density. However, Southeast Asia, the Indian subcontinent, and scattered hotspots in sub-Saharan Africa are at high risk due to the overlap between human populations and natural opportunities for cross-species transmission of betacoronaviruses.

Reassuringly, these predictions correspond to the geographic origins of the three bat-origin coronaviruses that have recently emerged in human populations. While available information puts the spillover of SARS-CoV-2 in a live animal market in Wuhan, China, the ultimate origin of the virus is almost certainly in a divergent lineage of sarbecoviruses from Indochina that was poorly characterized prior to the pandemic (Boni et al. 2020; Temmam et al. 2022; Worobey et al. 2022). Similarly, the SARS-CoV outbreak began in Guangdong province in 2002, reaching humans through small carnivore bridge hosts, but was eventually traced back to a set of likely progenitor viruses found in cave-dwelling horseshoe bats in Yunnan province (Hu et al. 2017) nearby, antibody evidence has indicated human exposure to SARS-like viruses (Wang et al. 2018). MERS-CoV was first detected in Jordan, but is widespread in camels in East Africa and the Middle East, and may have reached its bridge host decades earlier than originally supposed (Müller et al. 2014); as a result, the geography of the original bat-to-camel transmission is still widely regarded as uncertain. All of these are broadly consistent with the risk factors we identify. Notably, India and West Africa are additional hotspots that have yet to experience the emergence of a bat coronavirus into human populations, but may still be at risk—particularly given known gaps in bat surveillance (Cohen et al. 2022) and a dense population in both regions with global connectivity. In any of these regions, surveillance on viral reservoirs can be paired with targeted monitoring of high-risk human populations (i.e. those with regular wildlife contact) (Xu et al. 2004) for maximum impact.

## Conclusion

Bats emerged around 64 million years ago, and are one of the most diverse mammalian orders, with more than 1,400 estimated species (Peixoto, Braga, and Mendes 2018; Simmons and Cirranello 2020). They exhibit a broad variety of habitat use, behavior, and feeding strategies, putting them at key positions in the delivery and provisioning of several ecosystem services, tied to important ecosystem-derived benefits to humans (Kasso and Balakrishnan 2013). Over two-thirds of bats are known to be either obligate or facultative insectivores, therefore actively contributing for agricultural pest control, (Williams-Guillén, Perfecto, and Vandermeer 2008; Voigt and Kingston, 2016) and vectors of pathogens that put a risk on human health (Gonsalves et al. 2013a,b); some other species are essential links in many seed-dispersal networks (Mello et al. 2011). However, many of these species face a high risk of extinction, particularly given persecution and killings that sometimes follows from messaging about their role in disease emergence. Areas where bats, viruses, and humans co-occur are not always hotspots of risk for human heath; as such, developing more precise ways to map zoonotic hazards can help bats and humans coexist safely, and support the conservation of these important and unique animals.

Here, we propose a simple framework with broad explanatory power that helps contextualize discoveries like highly divergent nobecoviruses in Madagascar and the once-neglected adaptive radiation of sarbecoviruses in the Indochinese peninsula. In doing so, it advances ecological theory beyond the current state of the art for global maps of emergence risk. For example, previous studies that have used host richness as a proxy have predicted a high diversity of unsampled bat viruses (Olival et al. 2017), bat coronaviruses (Anthony et al. 2017), and even specifically betacoronaviruses (Becker et al. 2022) in both the Amazon and Southeast Asia. While we find that both regions are characterized by unique and diverse communities of both hosts and viruses, our framework is able to identify key differences between the two systems We find that the merbecovirus complex in Latin America has been a unique branch of evolution separate from the rest of the global pool, but with limited potential for viral diversification—a finding that is supported by previous work indicating a higher rate of codivergence in Latin America (Anthony et al. 2017; Caraballo 2022). In contrast, in southeast Asia, host richness and viral distinctiveness are high but sharing is low; this suggests a different type of evolutionary dynamics that could generate high local diversity of viruses through host switching and viral recombination (see e.g. Latinne et al. 2020) as well as the discovery of recombinant viruses with genetic material from both the SARS-CoV and SARS-CoV-2 branches of the Sarbecovirus lineage (Wu et al. 2021).^.^Both of these regions are priority areas for sampling, especially given predictions that they contain many bat hosts of undiscovered betacoronaviruses (Becker et al. 2022; Cohen et al. 2022). However, both the evolutionary and ecological aspects of emergence risk are higher in southeast Asia—a fact that will only become more relevant, as bats track shifting climates and exchange viruses with other species, creating a hotspot of elevated cross-species transmission unique to the region (Carlson et al. 2022; Muylaert et al. 2022).

Our trivariate additive mapping of components of risk (Fig. 3) aims to elicit the complexity of spatial cross-species transmission risk beyond the mere presence or absence of the pathogen host in a specific location. By considering coevolutionary factors such as viral sharing and host uniqueness, we suggest insights that can aid in identifying potential locations for surveillance of betacoronavirus circulation and assessing the risk of cross-species transmission to other mammals. In communities characterized by diverse but unique host populations, with limited viral sharing between them, we could encounter viruses that specialize in targeting the immune system of specific hosts. This implies a low likelihood of infecting novel hosts but, once locally introduced into a new host (either a new species, or an immunologically naïve population), the specialized virus could spread relatively easily due to encountering little immune resistance (Plowright et al. 2011). With the right combination of viral traits, such as low disease-induced mortality or high transmission rate, this could lead to successfully spread within the new host community. However, while high adaptation to a specific host can be advantageous, it may also lead to maladaptation when the pathogen encounters a new unsuitable host, potentially resulting in its extinction.

Bats—and the spillover of their viruses—are also sensitive to anthropogenic factors others than climate change, including deforestation and other kinds of habitat loss, increased stress, and greater contact with potential bridge hosts like domesticated species (Mendenhall et al. 2014; Treitler et al. 2016; Alves et al. 2018; Rulli et al. 2021). This represents a challenge for both conservation strategies and pandemic prevention (Amman et al. 2011) but identifying areas at risk, and protecting the health of bats and ecosystems within those zones, can be a win–win intervention for both (Hopkins et al. 2021^;^  Plowright et al. 2021; Adisasmito et al. 2022). As we scale these predictions down in space to finer spatial resolutions to guide public health actions (Muylaert et al. 2022), the incorporation of human activity predictors will become more importyant (Ka-Wai Hui 2006).

## Methods

### Known Betacoronavirus hosts

We downloaded the data on bats hosts of *Betacoronavirus* from https://www.viralemergence.org/betacov on Apr 2022 (Becker et al. 2022), and filtered it to ‘known’ hosts (established before the emergence of SARS-CoV-2) and ‘novel’ hosts (confirmed through sampling and competence assays since the initial data collection). The original database was assembled by a combination of data mining and literature surveys, including automated alerts on the ‘bats’ and ‘coronavirus’ keywords to identify novel empirical evidence of bats–betacoronaviruses associations; this yielded a total of 126 known hosts, 47 of which were novel hosts. This host–virus list of interactions was obtained through a comprehensive aggregation of GenBank data as well as systematic literature searches (Becker et al. 2022; Cohen et al. 2022), such that we have high confidence in its fitness for the purpose of inference at a large spatial scale.

### Bat occurrences

We downloaded the rangemap of every current bat species that was classified as an empirically documented host of *Betacoronavirus* from the previous step, according to recent IUCN data (IUCN 2021). The IUCN data have been assembled to support wildlife conservation efforts, and therefore we do not expect that they are biased by wildlife disease sampling efforts or priority. The range maps were subsequently rasterized using the rasterize function from GDAL (Rouault et al. 2022) at a resolution of ∼100 km ×100km at the equator. For every pixel in the resulting raster where at least one bat host of *Betacoronavirus* was present, we extract the species pool (list of all known bat hosts), which was used to calculate the following risk assessment components: bat phylogenetic diversity, bat compositional uniqueness, and predicted viral sharing risk.

### Bat phylogenetic diversity

For every pixel, we measured Faith’s Phylogenetic Diversity (Faith 1992) based on a recent synthetic tree with robust time calibration, covering about 6000 mammalian species (Upham, Esselstyn, and Jetz 2019). Faith’s PD measures the sum of unique branches from an arbitrary root to a set of tips, and comparatively larger values indicate a more phylogenetic diverse species pool. We measured phylogenetic diversity starting from the root of the entire tree (and not from Chiroptera); this bears no consequences on the resulting values, since all branches leading up to Chiroptera are only counted once per species pool, and (as we explain when describing the assembly of the composite risk map), all individual risk components are ranged in [0,1]. This measure incorporates a richness component, which we chose not to correct for; therefore, the interpretation of the phylogenetic diversity is a weighted species richness that accounts for phylogenetic over/under-dispersal in some places.

### Bat compositional uniqueness

For every species pool, we measured its Local Contribution to Beta-Diversity (LCBD) (Legendre and De Cáceres 2013) works from a species-data matrix (traditionally noted as **Y**), where species are rows and sites are columns, and a value of 1 indicates occurrence. We extracted the Y matrix assuming that every pixel represents a unique location, and following best practices (Legendre and Condit 2019) transformed it using Hellinger’s distance to account for unequal bat richness at different pixels. The correction of raw community data is particularly important for two reasons: first, it prevents the artifact of richer sites having higher importance; second, it removes the effect of overall species richness, which is already incorporated in the phylogenetic diversity component. High values of LCBD indicate that the pixel has a community that is, on an average, more dissimilar in species composition than what is expected knowing the entire matrix, i.e. a more unique community. Recent results by Dansereau, Legendre, and Poisot (2022) show that LCBD measures are robust with regards to spatial scale, and are therefore applicable at the global scale.

### Viral sharing between hosts

For all bat hosts of *Betacoronavirus*, we extracted their predicted viral sharing network, generated from a previously published generalized additive mixed model of virus sharing by a tensor function of phylogenetic distance and geographic range overlap across mammals (Albery et al. 2020). This network stores pairwise values of viral community similarity, measured for all hosts (to maintain consistency with the phylogenetic diversity measure) across all viruses; therefore, we consider that it accounts for some overall similarity in the way hosts deal with viruses, and not only betacoronaviruses. There is empirical evidence that capacity for cross-species transmission even between divergent species is generally high (Mollentze and Streicker 2020), especially for betacoronaviruses (Latinne et al. 2020). To project viral sharing values into a single value for every pixel, we averaged the pairwise scores. High values of the average sharing propensity means that this specific extant bat assemblage is likely to be proficient at exchanging viruses.

### Composite risk map

To visualize the aggregated risk at the global scale, we combine the three individual risk components (phylogenetic diversity, compositional uniqueness, and viral sharing) using an additive color model (Seekell, Lapierre, and Cheruvelil 2018). In this approach, every risk component gets assigned a component in the RGB color model (phylogenetic diversity is green, compositional uniqueness is red, and viral sharing is blue). In order to achieve a valid RGB measure, all components are re-scaled to the [0,1] interval, so that a pixel with no sharing, no phylogenetic diversity, and no compositional uniqueness is black, and a pixel with maximal values for each is white. This additive model conveys not only the intensity of the overall risk, but also the nature of the risk as colors diverge towards combinations of values for three risk components. Out of the possible combinations, the most risky in terms or rapid diversification and spillover potential is high phylogenetic diversity and low viral sharing (Gomulkiewicz et al. 2000) in that this allows multiple independent host–virus coevolutionary dynamics to take place in the same location. In the colorimetric space, this correspond to yellow—because the HSV space is more amenable to calculations for feature extraction (Keke, Peng, and Guohui 2010) we measured the risk level by calculating the angular distance of the hue of each pixel to a reference value of 60 (yellow), and weighted this risk level by the value component. Specifically, given a pixel with colorimetric coordinates (*h*, *s*, *v*), its ranged weighted risk value is


$$v\times\left[1-\frac{\left|\text{atan}\left(\text{cos}(\text{rad}(h)), \text{sin}(\text{rad}(h))\right) - X\right|}{2\pi}\right]\,,$$

where X is atan(cos(rad(60)),sin(rad(60))), a constant ≈ 0.5235.

### Viral phylogeography and evolutionary diversification

To next represent phylogeography of betacoronaviruses in bats, we aggregated and analyzed betacoronavirus sequence data. We used the following query to pull all *Betacoronavirus* sequence data from the GenBank Nucleotide database except SARS-CoV-2; (‘Betacoronavirus’[Organism] OR betacoronavirus[All Fields]) NOT (‘Severe acute respiratory syndrome coronavirus 2’[Organism] OR sars-cov-2[All Fields]). We added a single representative sequence for SARS-CoV-2 and manually curated to remove sequences without the RNA-dependent RNA polymerase (RdRp) sequence or that contained words indicating recombinant or laboratory strains including ‘patent’, ‘mutant’, ‘GFP’, and ‘recombinant’. We filtered over-represented taxa including betacoronavirus 1, hCoV-OC43, Middle East respiratory syndrome coronavirus, Murine hepatitis virus, and hCoV-HKU1. Curated betacoronavirus RdRp sequences were then aligned using MAFFT (Katoh and Standley et al. 2013) v1.4.0 (Algorithm FFT-NS-2, Scoring matrix 200PAM/k = 2, gap open penalty 1.53 metre offset value 0.123) and a maximum likelihood tree reconstructed in IQ-TREE (Nguyen et al. et al. 2015) v1.6.12 with ModelFinder (Kalyaanamoorthy et al. 2017) ultrafast bootstrap approximation (Hoang et al. 2018) with a general time reversible model with empirical base frequencies and the 5-discrete-rate-category FreeRaye model of nucleotide substitution (GTR + F + R5).

We first tested the hypothesis that hotspots of viral diversification would track hotspots of bat diversification. To do so, we plotted the number of known bat hosts (specifically only those included in the phylogeny, so there was a 1:1 correspondence between data sources) against the ‘mean evolutionary distinctiveness’ of the associated viruses. To calculate this, we derived the fair proportions evolutionary distinctiveness (Isaac et al. 2007) for each of the viruses in the tree, then averaged these at the bat species level, projected these values onto their geographic distributions, and averaged across every bat found in a given pixel. As such, this can be thought of as a map of the mean evolutionary distinctiveness of the known viral community believed to be associated with a particular subset of bats present.

### Co-distribution of hosts and viral hotspots

Subsequently, we tested the hypothesis that the biogeography of bat betacoronaviruses should track the biogeography of their hosts. To test this idea, we loosely adapted a method from, (Kreft and Jetz 2007^,^  2010) who proposed a phylogenetic method for the delineation of animal biogeographic regions. In their original method, a distance matrix—where each row or column represents a geographic raster’s grid cell, and the dissimilarity values are the ‘beta diversity similarity’ of their community assemble—undergoes non-metric multidimensional scaling (NMDS); the first two axes of the NMDS are projected geographically using a four-color bivariate map. Here, we build on this idea with an entirely novel methodology. First, we measure the phylogenetic distance between the different viruses in the betacoronaviruses tree by using the cophenetic function in ape; (Paradis and Schliep 2019) subsequently, we take a principal components analysis of that distance matrix (readily interchangeable for NMDS in this case) to project the viral tree into an *n*-dimensional space. We then take the first two principal components and, as with the evolutionary distinctiveness analysis, aggregated these to a mean host value and projected them using a four-color bivariate map.

## Data Availability

The code to reproduce these analyses, as well as the data (with the exception of the IUCN rangemaps, which must be downloaded from their website) are available in the *viralemergence/betamap* repository on GitHub.
